# A Smoothed Algorithm with Convergence Analysis under Generalized Maximum Correntropy Criteria in Impulsive Interference

**DOI:** 10.3390/e21111099

**Published:** 2019-11-11

**Authors:** Hua Qu, Youwei Shi, Jihong Zhao

**Affiliations:** 1 School of Electronic and Information Engineering, Xi’an Jiaotong University, Xi‘an 710049, China; qh@xjtu.edu.cn (H.Q.); zhaojihong@xjtu.edu.cn (J.Z.); 2 School of Telecommunication and Information Engineering, Xi’an University of Posts and Telecommunications, Xi’an 710061, China

**Keywords:** generalized correntropy, exponential weighted average, robust adaptive filtering algorithm, impulsive noise

## Abstract

The generalized maximum correntropy criterion (GMCC) algorithm is computationally simple and robust against impulsive noise but it suffers from slow convergence speed as it is derived and based on stochastic gradient, which only use the current data sample. In order to deal with this issue, a smoothed GMCC algorithm (SGMCC) is proposed. In the SGMCC algorithm, instead of taking the exponential weighted average of gradient vector to approximate the expectation of the gradient vector, we take the exponential weighted average of the variable step-size so that the SGMCC algorithm can be viewed as a sign GMCC algorithm with smoothed variable step-size. Moreover, convergence performance analyses are derived in terms of variable step-size, mean-square stability and steady-state behavior to demonstrate the robustness of the proposed algorithm. At last, simulation comparisons show that the proposed algorithm is robust against impulsive noise and converges fast with lower computational complexity. Also, for the steady-state behavior, simulation results verify that the simulated value matches well with the theoretical one.

## 1. Introduction

Information theoretic learning (ITL) [1] methods have been shown to be efficient approaches in non-Gaussian signal processing due to their robustness against impulsive noise. The maximum correntropy criterion (MCC) [2,3] is one of the most popular optimization criteria in ITL due to its simplicity and robustness. Recently, it has been successfully applied in various signal processing scenarios, particularly the adaptive filtering [4,5,6,7,8,9,10].

The introduction of the correntropy as a cost function into adaptive filters was proposed in Reference [11]. The theoretical analysis in Reference [12] has shown that the steady-state excess mean square error (EMSE) of the MCC algorithm is controlled by the step-size and the kernel width. Various kernel width selection methods for the MCC algorithm have been investigated. Adaptive kernel width adjusting methods were proposed in References [6,7] to improve the convergence speed of the MCC algorithm.

Just like the least mean square (LMS)-type algorithms, the MCC algorithm with fixed step-size is insufficient to achieve a good tradeoff between fast convergence and low steady-state misadjustment. The adaptive variable step-size technique is a promising way to deal with the conflicting requirements of faster learning speed and lower steady-state misadjustment error. Many varieties of variable step-size LMS-type algorithms have been proposed to improve the convergence performance [13,14,15,16]. However, many variable step-size methods based on instantaneous error cannot be directly applied in the MCC algorithm. Of course, just a few variable step-size methods for the MCC algorithm have been developed to improve the convergence performance in recent years. A convex combination of two MCC algorithms with different step-sizes was proposed in Reference [4]. The mixing factor in this method was adaptively updated so that the MCC algorithm with larger step-size can improve the convergence speed at the beginning while the other with smaller step-size can achieve a lower misadjustment at steady state. The combinational approach can achieve desirable convergence performance but its computational complexity was more than two times higher than that of the standard MCC algorithm since two MCC algorithms were adopted in parallel. A curvature based variable step-size method for the MCC algorithm was proposed in Reference [17]. This developed method can improve the convergence speed at the initial stage especially when the weight vector is far away from the optimal solution.

In recent years, a generalized maximum correntropy criterion (GMCC) has been proposed, which adopts the generalized Gaussian density [18,19,20] function as the kernel function (not necessarily a Mercer kernel [21]) and the type of this correntropy is called the generalized correntropy. Similar to the original correntropy with Gaussian kernel, the generalized correntropy can also be used as an optimization cost in the estimation-related problems. Under the GMCC criterion, a stochastic-gradient based adaptive filtering algorithm, called the GMCC algorithm, was developed [21]. We can see that the GMCC algorithm was derived and based on stochastic gradient which only uses the current data sample. Although the GMCC algorithm is computationally simple and robust in the presence of impulsive noise, it suffers from high steady-state mean square deviation (MSD), which also can be verified from the next simulation.

In this paper, a smoothed GMCC algorithm (called SGMCC) is proposed to improve the performance of steady-state MSD in the presence of impulsive noise. To avoid the high steady-state MSD caused by the stochastic gradient in the GMCC algorithm, we take the GMCC algorithm as a sign algorithm with variable step-size. Then, we take the exponential weighted average of the variable step-size rather than the gradient vector, which contributes to reduce the computational complexity and further improve the convergence speed. At last, we present the convergence performance analyses of the proposed algorithm to demonstrate its robustness. The main contributions of our paper can be summarized as follows:

1. We propose a novel SGMCC algorithm, which can improve the performance of steady-state MSD and is robust against impulsive noise.
2. To demonstrate the robustness of the proposed algorithm, we derive the convergence performance analyses from three aspects including the variable step-size, mean-square stability and steady-state behavior.
3. Simulation results demonstrate that the proposed algorithm is robust against impulsive noise and computationally simple. Also, the SGMCC algorithm owns faster convergence speed compared with other robust adaptive algorithms.

The rest of the paper is organized as follows. In Section 2, after a brief review of the original GMCC algorithm, a SGMCC algorithm is proposed. In Section 3, the convergence performances of the SGMCC algorithm are studied. In Section 4, Monte-Carlo simulation results are presented to confirm the desirable performance of the proposed algorithm. Finally, a conclusion is given in Section 5.

## 2. SGMCC Algorithm

Assume the received signal $d_{n}$ is obtained from an unknown system $W_{*}$

(1) $$d_{n}=W_{*}^{T}X_{n}+v_{n}$$

where $y_{n}=W_{*}^{T}X_{n}$ denotes the output of the unknown system, $X_{n}$ is the M×1 dimension input signal vector at *n*th time index and $W_{*}$ denotes the M×1 dimension unknown system vector that we wish to estimate. Here, ${\left(\cdot \right)}^{T}$ means the vector transpose operator.

In Equation (1), $v_{n}$ denotes the Gaussian mixture distribution noise, which is modeled as a random variable of 2-components Gaussian mixture distribution with mixed coefficient $p_{r}$. Its probability density function can be expressed as follows:

(2) $$MG\left(\sigma_{1},\sigma_{2},p_{r}\right)=\left(1-p_{r}\right)N\left(0,\sigma_{1}^{2}\right)+p_{r}N\left(0,\sigma_{2}^{2}\right)$$

where the first Gaussian distribution $N(0,\sigma_{1}^{2})$ generates the normal Gaussian noise with probability $\left(1-p_{r}\right)$, the second Gaussian distribution $N(0,\sigma_{2}^{2})$ generates impulsive noise with probability $p_{r}$. Usually, $\sigma_{1}\ll \sigma_{2}$ and the impulsive noise occurrence probability $p_{r}$ is set as a number that is near but larger than 0. $\sigma_{1}^{2}$ and $\sigma_{2}^{2}$ denotes the Gaussian noise variance and the impulsive noise variance, respectively.

### 2.1. Review of the GMCC Algorithm

As it is well-known that MCC has been successfully applied in various non-Gaussian signal processing matters due to its robustness properties [5,6,8,22]. As a non-linear local similarity measure between two random variables *x* and *y*, the correntropy is defined by [2,3]:

(3) $$V_{\sigma }\left(x,y\right)=E\left[\kappa_{\sigma }\left(x,y\right)\right]$$

where $\kappa_{\sigma }\left(\cdot \right)$ denotes a shift-invariant Mercer kernel. The kernel function is a key factor of the correntropy that dramatically affects the similarity of two random variables. In most case, the Gaussian density function is used as kernel function, which is defined as follows:

(4) $$\kappa_{\sigma }\left(x,y\right)=\exp\left(-\frac{{\left|x-y\right|}^{2}}{\sigma^{2}}\right)$$

However, for all non-Gaussian signal processing applications, the Gaussian density function is not always the best kernel function. In order to deal with this issue and take full advantage of the potential robustness properties of the correntropy, the generalized Gaussian density function was proposed as a kernel function of the correntropy in Reference [21]. The generalized Gaussian density function with zero-mean is given by References [18,19] as follows:

(5) Gα,β(x,y)=α2βΓ11ααexp−x−yβα

where $\Gamma \left(\cdot \right)$ is the gamma function, α>0 denotes the shape parameter and β>0 denotes the scale parameter. For simplicity, α is usually set as an integer value. Note that the Gaussian kernel is a special case of the generalized Gaussian density function when α=2.

In practice, the distribution of the data is usually unknown and only a finite number of samples are available. It is common to use these samples of the data to approximate its expectation, so the generalized corretropy can be estimated by [21]:

(6) $${\hat{V}}_{\alpha ,\beta }\left(X,Y\right)=\frac{1}{N}\sum_{i=1}^{N}G_{\alpha ,\beta }\left(x_{i},y_{i}\right)$$

Just like the correntropy, the generalized correntropy can be used as a cost function in adaptive filters. Hence, the generalized correntropy between the desired signal $d_{n}$ and the filter output $y_{n}$ can be shown as a cost function by [21]:

(7) $$J_{GMCC}=E\left[G_{\alpha ,\beta }\left(e_{n}\right)\right]=\gamma_{\alpha ,\beta }E\left[\exp\left(-\lambda {\left|e_{n}\right|}^{\alpha }\right)\right]$$

where $e_{n}=d_{n}-y_{n}$ is the instantaneous prediction error, γα,β=αα2βΓ11αα2βΓ11αα represents the normalization constant and $\lambda =\beta^{-\alpha }$ denotes the kernel parameter.

The goal of the adaptive filtering algorithm is to find an estimator of the unknown system vector which maximizes the generalized correntropy of $e_{n}$. The optimal solution of this cost maximization problem can be solved by a stochastic-gradient based adaptive algorithm, called GMCC algorithm [21]. The update expression of the weight vector in the GMCC algorithm can be derived as [21]

(8) $$W_{n+1}=W_{n}+\eta \exp\left(-\lambda {\left|e_{n}\right|}^{\alpha }\right){\left|e_{n}\right|}^{\alpha -1}\mathrm{sign}\left(e_{n}\right)X_{n}$$

where η is the step-size parameter and sign() is a universal sign function. It is obvious that the GMCC algorithm can be viewed as a sign algorithm with variable step-size $\eta \exp\left(-\lambda {\left|e_{n}\right|}^{\alpha }\right){\left|e_{n}\right|}^{\alpha -1}$.

### 2.2. SGMCC Algorithm

As mentioned above, although the GMCC algorithm is robust and computationally simple, it suffers from high steady-state MSD as it is derived and based on stochastic gradient which only uses the current data sample. The instantaneous prediction error $e_{n}$ fluctuates randomly with the background noise $v_{n}$ and the input vector $X_{n}$. The random fluctuations of $e_{n}$ and $X_{n}$ can disturb the update of the weight vector and thus lead to a slow convergence speed.

To deal with this issue, it is common practice to take the average of the latest *N* sample data to approximate the expectation of the gradient vector, which can statistically reduce the adverse effects caused by the randomness of $e_{n}$ and $X_{n}$. Hence, the update expression of the weight vector in Equation (8) can be rewritten as follows:

(9) $$W_{n+1}=W_{n}+\frac{\eta }{N}\sum_{i=n-N+1}^{n}\exp\left(-\lambda {\left|e_{i}\right|}^{\alpha }\right){\left|e_{i}\right|}^{\alpha -1}\mathrm{sign}\left(e_{i}\right)X_{i},$$

where $e_{i}=d_{i}-W_{n}^{T}X_{i}$ is the prediction error at *n*th iteration for the input vector $X_{i}$. It is worth noting that the calculation of each instantaneous prediction error $e_{i}\;\left(i=n-N+1,...,n\right)$ is based on the present weight vector $W_{n}$, so the weight update in Equation (9) needs *N* times of computational cost and storage cost than that of Equation (8).

In order to reduce the computational cost and the storage cost, the exponential weighted average of the gradient vector can be adopted to approximate the expectation of the gradient vector, which is shown as follows:

(10) $${\bar{p}}_{n}=\theta {\bar{p}}_{n-1}+\left(1-\theta \right)\exp\left(-\lambda {\left|e_{n}\right|}^{\alpha }\right){\left|e_{n}\right|}^{\alpha -1}\mathrm{sign}\left(e_{n}\right)X_{n},$$

where ${\bar{p}}_{n}$ is the exponential weighted average of the GMCC gradient vector with a smoothing factor θ(0<θ<1). In this case, the update expression of the weight vector can be further expressed as

(11) $$W_{n+1}=W_{n}+\eta {\bar{p}}_{n}.$$

Although computational cost of Equation (11) is much lower than that of Equation (9), it still needs extra *N* multiplications and additions than that of Equation (8). In order to reduce the computational cost derived from gradient vector, we take the GMCC algorithm as a variable step-size sign algorithm by taking the exponential weighted average of the variable step-size instead of that of gradient vector. The exponential weighted average of the variable step-size, $\mu_{n}$, can be expressed as follows:

(12) $$\mu_{n}=\theta \mu_{n-1}+\left(1-\theta \right)\exp\left(-\lambda {\left|e_{n}\right|}^{\alpha }\right){\left|e_{n}\right|}^{\alpha -1}.$$

The replacement of the variable step-size from gradient vector can further reduce the computational cost and lead to a smoothed GMCC algorithm, namely SGMCC. The weight vector update of the SGMCC algorithm can be expressed as follows:

(13) $$W_{n+1}=W_{n}+\eta \mu_{n}\mathrm{sign}\left(e_{n}\right)X_{n}.$$

For convenience, the proposed SGMCC algorithm is specifically summarized in Algorithm 1.


**Algorithm 1**   SGMCC Algorithm.
Input: $X_{n}$, $d_{n}$, α, λ, θ, η.
Initialize: $W_{1}=0$, $e_{0}=d_{1}$, $\mu_{0}=0$.
   **while**
$\left\{X_{n},d_{n}\right\}$ available
      $e_{n}=d_{n}-W_{n}^{T}X_{n}$,
      $\mu_{n}=\theta \mu_{n-1}+\left(1-\theta \right)\exp\left(-\lambda {\left|e_{n}\right|}^{\alpha }\right){\left|e_{n}\right|}^{\alpha -1}$ according to Equation (12),
      $W_{n+1}=W_{n}+\eta \mu_{n}\mathrm{sign}\left(e_{n}\right)X_{n}$ according to Equation (13),
   **end while**

   $W_{*}=W_{n+1}$,
Output: estimated unknown system $W_{*}$.

### 2.3. Computational Complexity

The complexity of the adaptive filtering algorithm is one of the important factors to measure its performance. The recursive weight updates of adaptive filtering algorithm generally include addition and multiplication operations. Since the complexity of multiplication operations is much higher than that of additive operations, in the iterative update process, the number of multiplication operations is usually used to calculate the computational complexity of the adaptive filtering algorithm. Next, we will make a comparison of the computational complexity between SGMCC and several other robust algorithms. The detail is summarized in Table 1.

As one can see from Table 1, the computational complexity of the proposed SGMCC algorithm and the GMCC algorithm are much lower than that of the other algorithms. The SGMCC algorithm owns almost the same the computational complexity as the GMCC algorithm. Those two algorithms require about 2M+α (usually α<M) multiplications, which is just about half of that required by other robust algorithms. The CMCC algorithm requires the most multiplication operations since it has to calculate two MCC algorithms with different step-sizes and update the combination parameter to combine those two MCC algorithms. The VSSA algorithm still requires more multiplication operations than the proposed SGMCC algorithm as this algorithm involves the calculation of the weighted average of the gradient vector and its square norm.

## 3. Convergence Performance Analysis

### 3.1. Analysis of the Variable Step-Size

Since the proposed SGMCC algorithm can be viewed as a variable step-size sign algorithm, it is necessary to evaluate the variable step-size theoretically. For the proposed SGMCC algorithm, the initial value of the variable step size is usually set as $\mu_{0}=0$, so the variable step size can be expressed as

(14) $$\mu_{n}=\left(1-\theta \right)\sum_{i=1}^{n}\theta^{n-i}\exp\left(-\lambda {\left|e_{i}\right|}^{\alpha }\right){\left|e_{i}\right|}^{\alpha -1}.$$

Equation (14) shows the transient value of the variable step-size. If the transient variable step-size is positive and bounded, the adaptive algorithm can converge to steady state. It is obvious that $\mu_{n}$ is positive, namely

(15) $$\mu_{n}=\left(1-\theta \right)\sum_{i=1}^{n}\theta^{n-i}\exp\left(-\lambda {\left|e_{i}\right|}^{\alpha }\right){\left|e_{i}\right|}^{\alpha -1}>0.$$

In order to get the upper bound of the variable step size, we take the derivative of $\phi \left(e_{i}\right)=\exp\left(-\lambda {\left|e_{i}\right|}^{\alpha }\right){\left|e_{i}\right|}^{\alpha -1}$ with respect to $e_{i}$ and set it to 0, namely

(16) $$\frac{\partial \phi \left(e_{i}\right)}{\partial e_{i}}=\exp\left(-\lambda {\left|e_{i}\right|}^{\alpha }\right){\left|e_{i}\right|}^{\alpha -2}\left[\left(\alpha -1\right)-\lambda \alpha {\left|e_{i}\right|}^{\alpha }\right]=0,$$

then we can obtain the variable $e_{i}$ which maximizes $\phi \left(e_{i}\right)$:

(17) $${\left|e_{i}\right|}^{\alpha }=\frac{\alpha -1}{\lambda \alpha }$$

so the maximum of $\phi \left(e_{i}\right)$ is

(18) $$\max\phi \left(e_{i}\right)={\left(\frac{\alpha -1}{\lambda \alpha }\right)}^{\frac{\alpha -1}{\alpha }}\exp\left(-\frac{\alpha -1}{\alpha }\right).$$

Usually the shape parameter α is an integer (larger than 1) and the kernel parameter λ<1, so we have

(19) $$\max\phi \left(e_{i}\right)={\left(\frac{\alpha -1}{\lambda \alpha }\right)}^{\frac{\alpha -1}{\alpha }}\exp\left(-\frac{\alpha -1}{\alpha }\right)<\lambda^{-1}.$$

Above all, we can get the range of the variable step-size

(20) $$0<\mu_{n}<\lambda^{-1}.$$

We can see that the variable step size of the proposed algorithm is positive and bounded, so the proposed SGMCC algorithm can converge to steady state when a suitable step-size parameter η is selected. When the adaptive algorithm converges to steady state, the variable step-size converges to a constant, which determines the steady-state accuracy of the adaptive filtering algorithm.

After taking the expectation for both sides of Equation (14), we obtain

(21) $$E\left[\mu_{n}\right]=\left(1-\theta \right)E\left[\sum_{i=1}^{n}\theta^{n-i}\exp\left(-\lambda {\left|e_{i}\right|}^{\alpha }\right){\left|e_{i}\right|}^{\alpha -1}\right].$$

When the step-size parameter η is small enough, the weight vector $W_{n}$ can approach very close to the optimal weight vector $W_{*}$ and the instantaneous prediction error $e_{n}$ is dominated by the background noise $v_{n}$. Assume the proposed SGMCC algorithm converges to steady state when n>N, Equation (21) can be approximated as

(22) $$\lim_{n\to N}E\left[\mu_{n}\right]=\left(1-\theta \right)E\left[\lim_{n\to N}\sum_{i=1}^{n}\theta^{n-i}\exp\left(-\lambda {\left|v_{i}\right|}^{\alpha }\right){\left|v_{i}\right|}^{\alpha -1}\right].$$

The background noise $v_{n}$ is usually assumed to be independent and identically distributed, so Equation (22) can be denoted as

(23) $$\lim_{n\to N}E\left[\mu_{n}\right]=\left(1-\theta \right)\lim_{n\to N}\sum_{i=1}^{n}\theta^{n-i}E\left[\exp\left(-\lambda {\left|v_{i}\right|}^{\alpha }\right){\left|v_{i}\right|}^{\alpha -1}\right].$$

When *N* is large enough, Equation (23) can be further simplified as

(24) $$E\left[\mu_{N}\right]=E\left[\exp\left(-\lambda {\left|v_{N}\right|}^{\alpha }\right){\left|v_{N}\right|}^{\alpha -1}\right].$$

### 3.2. Mean-Square Stability

The energy conservation relation (ECR) is the most widely used method to evaluate the convergence performance analysis of an adaptive filtering algorithm [12,16,21,23,24,25]. In this paper, we also use the ECR analysis to successively derive the mean-square stability and the steady-state behavior of the proposed SGMCC algorithm.

For the simplicity of the ECR analysis, the recursive weight update of an adaptive filtering algorithm can be denoted as 

(25) $$W_{n+1}=W_{n}+\eta f\left(e_{n}\right)X_{n}.$$

For the proposed SGMCC algorithm, $f(e_{n})$ can be expressed as

(26) $$f\left(e_{n}\right)=\mu_{n}\mathrm{sign}\left(e_{n}\right),$$

where the instantaneous prediction error $e_{n}$ can be denoted as

(27) $$e_{n}=d_{n}-W_{n-1}^{T}X_{n}=e_{a,n}+v_{n},$$

where $e_{a,n}={{\tilde{W}}_{n}}^{T}X_{n}$ is an a priori error. Here, ${\tilde{W}}_{n}=W_{*}-W_{n}$ represents the weight-error vector.

The performance of the adaptive filtering algorithm is usually measured by the MSD value of the weight-error vector ${\tilde{W}}_{n}$, namely

(28) $$MSD=E\left[{∥{\tilde{W}}_{n}∥}^{2}\right].$$

The weight-error vector at *n*th iteration can be derived as

(29) $${\tilde{W}}_{n+1}={\tilde{W}}_{n}+\eta f\left(e_{n}\right)X_{n}.$$

For both sides of Equation (29), we successively take the squared-norms and expectations, then we obtain the ECR expression as follows:

(30) $$E\left[{∥{\tilde{W}}_{n+1}∥}^{2}\right]=E\left[{∥{\tilde{W}}_{n}∥}^{2}\right]-2\eta E\left[f\left(e_{n}\right)e_{a,n}\right]+\eta^{2}E\left[f^{2}\left(e_{n}\right){∥X_{n}∥}^{2}\right].$$

To facilitate the convergence analysis of the SGMCC algorithm based on the energy conservation relationship, some commonly-used assumptions [12,16,21,23,24,25] are listed:

**Assumption** **1** (A1)**.** *The background noise sequence $\left\{v_{n}\right\}$ is independent and identically distributed (i.i.d.). Also it is independent of the input vector sequence $\left\{X_{n}\right\}$.*

**Assumption** **2** (A2)**.** *The filter is long enough such that $e_{a,n}$ is zero-mean Gaussian and independent of the background noise $v_{n}$.*

**Remark** **1.** *A1 is a valid assumption for most practical applications and is a basic assumption often used in signal processing. Moreover, it should be noted that, unlike most conventional signal processing methods that assume the background noise $v_{n}$ is Gaussian distribution, A1 does not impose any restrictions on the statistical distribution of the background noise. Based on A1, it is easy to conclude that the a priori error $e_{a,n}$ and background noise $v_{n}$ are independent, which is assumed by A2. According to the Central Limit Theorem, it is reasonable that the a priori error $e_{a,n}$ satisfies the Gaussian distribution.*

Mean-square stability analysis is carried out to determine the upper bound of step-size parameter η that makes sure the SGMCC algorithm is convergent. The adaptive algorithm is convergent implies that MSD is monotonously decreasing. That is to say that the following condition exists:

(31) $$E\left[{∥{\tilde{W}}_{n+1}∥}^{2}\right]\leq E\left[{∥{\tilde{W}}_{n}∥}^{2}\right]$$

Based on the ECR relation, the following inequality holds:

(32) $$2E\left[f\left(e_{n}\right)e_{a,n}\right]\geq \eta E\left[f^{2}\left(e_{n}\right){∥X_{n}∥}^{2}\right].$$

So, the step-size parameter η that makes adaptive filtering algorithm converges to steady state would be

(33) $$\eta \leq 2\underset{n\geq 0}{\inf}\frac{E\left[f\left(e_{n}\right)v_{n}\right]}{E\left[{\left|f\left(e_{n}\right)\right|}^{2}{∥X_{n}∥}_{2}^{2}\right]}.$$

In our case, the scale parameter η that ensures the stability of the SGMCC algorithm should meet

(34) $$\eta \leq 2\underset{n\geq 0}{\inf}\frac{E\left[\mu_{n}\mathrm{sign}\left(e_{n}\right)e_{a,n}\right]}{E\left[\mu_{n}^{2}{∥X_{n}∥}_{2}^{2}\right]}.$$

Because $\mu_{n}$ is an exponential weighted average of $\exp\left(-\lambda {\left|e_{i}\right|}^{\alpha }\right){\left|e_{i}\right|}^{\alpha -1}$, so the correlation between $\mu_{n}$ and $e_{n}$, $e_{a,n}$ is negligible when the smoothing factor β is close to 1. Therefore, the inequality Equation (34) can be further rewritten as follows:

(35) $$\eta \leq 2\underset{n\geq 0}{\inf}\frac{E\left[\mu_{n}\right]E\left[\mathrm{sign}\left(e_{n}\right)e_{a,n}\right]}{E\left[\mu_{n}^{2}\right]E\left[{∥X_{n}∥}_{2}^{2}\right]}.$$

Next, considering the above mentioned condition $0<\mu_{n}<\lambda^{-1}$, we obtain a smaller lower limit of the step-size parameter η:

(36) $$\eta \leq \frac{2\lambda }{\mathrm{Tr}\left(\mathrm{Rx}\right)}\underset{n\geq 0}{\inf}E\left[\mathrm{sign}\left(e_{n}\right)e_{a,n}\right],$$

where $\mathrm{Rx}=E\left[X_{n}{X_{n}}^{H}\right]$ is the covariance matrix of the input vector and Tr(·) denotes the trace operator.

In this paper, the impulsive background noise $v_{n}$ is modeled as a random variable that follows a 2-component Gaussian mixture distribution $MG\;\left(\;\sigma_{1},\;\sigma_{2},\;p_{r}\;\right)$. Based on the assumption A2, the Price’s theorem [26] and References [16,27], we have

(37) $$E\left[\mathrm{sign}\left(e_{a,n}\;+\;v_{n}\right)e_{a,n}\right]\;\;=\;\;E\left[e_{a,n}^{2}\right]\sqrt{\frac{2}{\pi }}\;\;\left\{\;\;\frac{1-p_{r}}{\sqrt{E\left[e_{a,n}^{2}\right]\;\;+\;\;\sigma_{1}^{2}}}\;+\;\frac{p_{r}}{\sqrt{E\left[e_{a,n}^{2}\right]\;\;+\;\;\sigma_{2}^{2}}}\;\;\right\}.$$

The Cramer-Rao [28] bound *c* is the minimum mean square error that the a priori error $e_{a,n}$ of an adaptive filtering algorithm can reach, so the step-size parameter η for the SGMCC algorithm would be

(38) $$\eta \leq \frac{2\lambda c}{\mathrm{Tr}\left(\mathrm{Rx}\right)}\sqrt{\frac{2}{\pi }}\left\{\frac{1-p_{r}}{\sqrt{c+\sigma_{1}^{2}}}+\frac{p_{r}}{\sqrt{c+\sigma_{2}^{2}}}\right\}.$$

### 3.3. Steady-State Behavior

The steady state behavior is usually measured by $E\left[e_{a,n}^{2}\right]$ when the adaptive filtering algorithm converges to steady state. The steady-state value of $E\left[e_{a,n}^{2}\right]$ is generally known as EMSE. Assume the adaptive filtering algorithm converges to steady state when n>N, then EMSE can be denoted as

(39) $$S=\lim_{n\to N}E\left[e_{a,n}^{2}\right].$$

MSD would converge to a constant when the algorithm reaches steady-state, so we have

(40) $$\lim_{n\to N}E\left[{∥{\tilde{W}}_{n+1}∥}^{2}\right]=\lim_{n\to N}E\left[{∥{\tilde{W}}_{n}∥}^{2}\right].$$

Based on the ECR relation, we have

(41) $$2\lim_{n\to N}E\left[f\left(e_{n}\right)e_{a,n}\right]=\eta \lim_{n\to N}E\left[f^{2}\left(e_{n}\right){∥X_{n}∥}^{2}\right].$$

For the proposed SGMCC algorithm, Equation (41) can be expressed as

(42) $$2\lim_{n\to N}E\left[\mu_{n}\mathrm{sign}\left(e_{n}\right)e_{a,n}\right]=\eta \lim_{n\to N}E\left[\mu_{n}^{2}{∥X_{n}∥}^{2}\right].$$

With the same reason mentioned above, the correlation between $\mu_{n}$ and $e_{n}$, $e_{a,n}$ is negligible. Therefore, the following expression holds

(43) $$2\lim_{n\to N}E\left[\mu_{n}\right]E\left[\mathrm{sign}\left(e_{n}\right)e_{a,n}\right]=\eta \mathrm{Tr}\left(\mathrm{Rx}\right)\lim_{n\to N}E\left[\mu_{n}^{2}\right].$$

As is derived in Equation (24), $E\left[\mu_{N}\right]$ would converge to a constant when the η is small enough. Therefore, Equation (43) becomes

(44) $$2\lim_{n\to N}E\left[\mathrm{sign}\left(e_{n}\right)e_{a,n}\right]=\eta \mathrm{Tr}\left(\mathrm{Rx}\right)E\left[\mu_{N}\right].$$

The impulsive background noise is assumed to be Gaussian mixture noise that follows $MG\;\left(\;\sigma_{1},\;\sigma_{2},\;p_{r}\;\right)$. Based on the assumption A2, the Price’s theorem [26] and References [16,27], we have

(45) $$\lim_{n\to N}E\left[\mathrm{sign}\left(e_{a,n}+v_{n}\right)e_{a,n}\right]=S\sqrt{\frac{2}{\pi }}\left\{\frac{1-p_{r}}{\sqrt{S+\sigma_{1}^{2}}}+\frac{p_{r}}{\sqrt{S+\sigma_{2}^{2}}}\right\}.$$

After substituting Equations (44) and (45) into Equation (43), we obtain

(46) $$S=\sqrt{\frac{\pi }{8}}\eta \mathrm{Tr}\left(\mathrm{Rx}\right)E\left[\mu_{N}\right]{\left\{\frac{1-p_{r}}{\sqrt{S+\sigma_{1}^{2}}}+\frac{p_{r}}{\sqrt{S+\sigma_{2}^{2}}}\right\}}^{-1}.$$

When the step-size parameter η is small enough, EMSE or *S* would converge to a smaller number, which is negligible comparing with the power of background noises. In this case, Equation (46) can be approximated by

(47) $$S\approx \eta \mathrm{Tr}\left(\mathrm{Rx}\right)E\left[\mu_{N}\right]\sqrt{\frac{\pi }{8}}\frac{\sigma_{2}\sigma_{1}}{\sigma_{2}\left(1-p_{r}\right)+\sigma_{1}p_{r}}.$$

Note that the theoretical EMSE value computed by (Equation (47)) is an approximate value that the SGMCC algorithm can achieve at steady state. The accuracy of the approximation is largely affected by the step-size parameter η. A valid approximation of EMSE can be secured with a small-enough η.

Usually, $\sigma_{1}\ll \sigma_{2}$ and $p_{r}\ll 1-p_{r}$, Equation (47) can be simplified as

(48) $$S\approx \eta \mathrm{Tr}\left(\mathrm{Rx}\right)E\left[\mu_{N}\right]\sqrt{\frac{\pi }{8}}\frac{\sigma_{1}}{1-p_{r}}.$$

## 4. Simulation Results

To validate the theoretical analysis and evaluate the performance of the proposed SGMCC algorithm, we conduct simulations in a channel estimation [29,30,31,32] with Gaussian mixture background noise. An unknown time-varying channel with 20 multipath subchannels is randomly generated and then normalized by $W_{opt}^{H}W_{opt}=1$. The input signals are generated by a zero-mean Gaussian distribution with unit variance. The input signals are transmitted via the above mentioned unknown channel and contaminated by impulsive background noise generated by a 2-component Gaussian mixture distribution $MG\left(\sigma_{1},\sigma_{2},p_{r}\right)$. The simulation results are averaged over 100 independent Monte-Carlo experiments.

**Figure 1:**
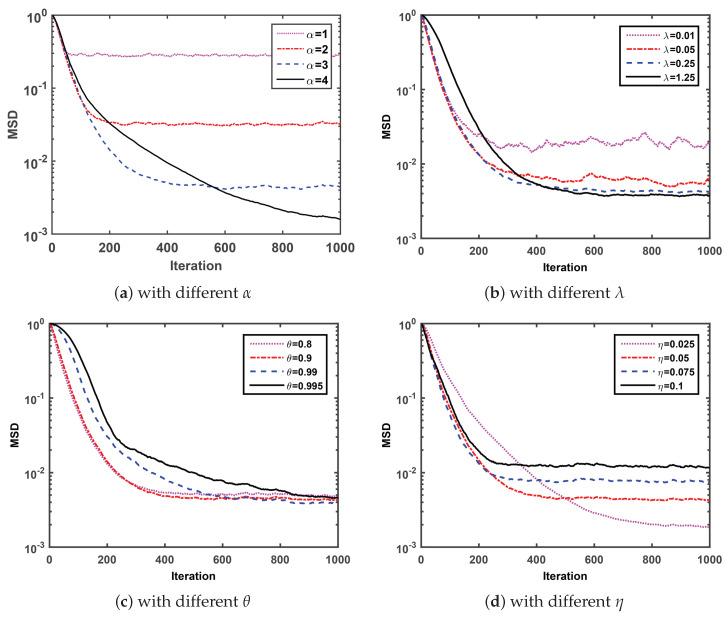
MSD convergence curves of SGMCC algorithm.

Firstly, we compare the MSD convergence curves of the proposed SGMCC algorithm with four parameters and investigate the effect of each parameter on the convergence performance of the SGMCC algorithm. Note that Figure 1 is obtained with $\sigma_{1}=0.2$, $\sigma_{2}=100$ and $p_{r}=0.1$. At each simulation, we set different values for a specific parameter with the other three parameters fixed and observe the effect of the parameter on the corresponding MSD convergence curve.

Under the condition of fixed parameter λ=0.25, θ=0.9 and η=0.05, Figure 1a shows that the SGMCC algorithm with larger shape parameter α converges slower to a lower steady-state MSD. As one can see in Figure 1b, under the condition of fixed parameter α=3, θ=0.9 and η=0.05, the MSD convergence curve of SGMCC algorithm with smaller kernel parameter λ is not smooth and fluctuates around a larger range. However, a larger kernel parameter λ may lead to a slower convergence speed. As observed in Figure 1c, under the condition of fixed parameter α=3, λ=0.25 and η=0.05, the SGMCC algorithm with different smooth factor θ values converges to the almost same steady-state MSD after 1000 iterations. In addition, the SGMCC algorithm with smaller θ converges faster but the improvement of the convergence speed is not obvious when θ smaller than 0.9. At last in Figure 1d, under the condition of fixed parameter α=3, λ=0.25 and θ=0.9, the SGMCC algorithm with smaller step-size parameter η can achieve lower steady-state MSD at the cost of a slower convergence speed and vice versa.

Secondly, we compare the convergence MSD performance of our proposed algorithm with several recently published algorithms to demonstrate its convergence performance. The parameters of the GMCC algorithm is set the same as that of the SGMCC algorithm. The parameters of other algorithms are carefully adopted according to the corresponding reference. The compared algorithms and their corresponding parameters were summarized in Table 2.

**Figure 2:**
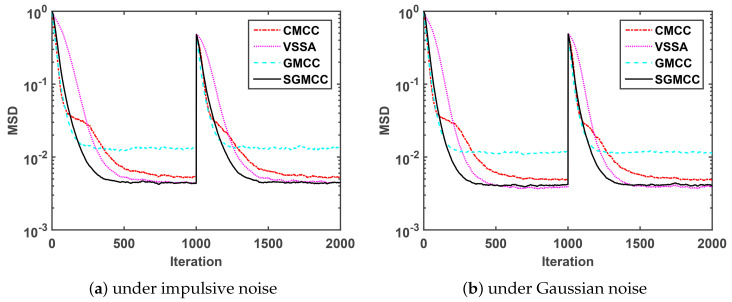
Comparison of the mean square deviation (MSD) curves versus iteration.

Figure 2a shows a comparison of the MSD convergence curves of different algorithms under impulsive noise generated by $MG\left(0.2,100,0.1\right)$. As observed in Figure 2a, the proposed SGMCC algorithm achieves a much lower steady-state MSD than the GMCC algorithm with the same parameters. The SGMCC algorithm and the VSSA algorithm converge to the same and the lowest steady-state MSD. On the other hand, the proposed SGMCC algorithm converges faster than the VSSA algorithm and the CMCC algorithm. Different from Figure 2a simulated under impulsive noise, Figure 2b is conducted under Gaussian noise. We can see that Figure 2b shows the similar results with Figure 2a. So we can conclude that, compared with the other three algorithms, the proposed SGMCC algorithm achieves the lowest steady-state MSD with a relatively fast convergence speed. Also, the proposed SGMCC algorithm is robust against both impulsive noise and Gaussian noise.

**Figure 3:**
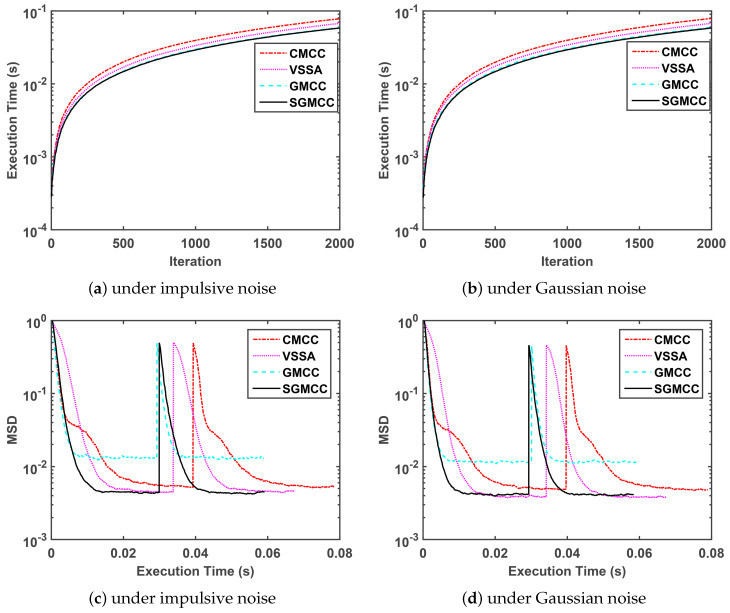
Comparison of the execution time.

Thirdly, we compare the execution time of our proposed algorithm with several recently published algorithms in the next Figure 3. The parameter setting of four algorithms in Figure 3 is same with that in Figure 2, which is shown in Table 2. Note that the measured execution time of four algorithms is related to the running platform and specific code-programming efficiency.

Figure 3a,b shows a comparison of the execution time curves of different algorithms versus iteration under impulsive noise and Gaussian noise, respectively, and these two subfigures show the same performance trend. Figure 3c,d shows a comparison of the steady-state MSD curves of different algorithms versus execution time under impulsive noise and Gaussian noise respectively and these two subfigures show the same performance trend. Concretely speaking, Figure 3a,b demonstrate that the proposed SGMCC algorithm owns the shortest run time and is close to the GMCC algorithm. Figure 3c,d demonstrate that the SGMCC algorithm owns the shortest run time by observing the length of the curve tail. Although the run time of the proposed SGMCC algorithm is also is close to that of the GMCC algorithm, the SGMCC algorithm achieves a much lower steady-state MSD. Moreover, the superiority of the SGMCC algorithm in run time can be ensured by computational complexity shown in Table 1.

**Figure 4:**
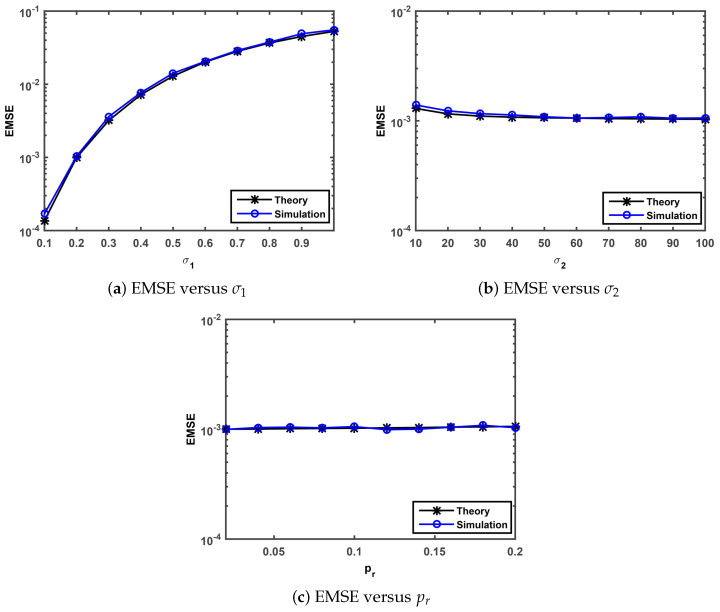
Comparison of the theoretical result with the simulation result.

Finally, we perform simulations to confirm the steady-state behavior analysis presented in Section 3.3. The theoretical steady-state EMSEs are evaluated by Equation (47). The simulated steady state EMSEs were calculated as a average over the last 500 iterations of EMSE that is averaged over 50 independent Monte-Carlo simulation with 5000 iterations. We investigate the theoretical and simulated EMSEs under $MG\;\left(\sigma_{1},\sigma_{2},p_{r}\right)$ versus Gaussian noise standard deviation $\sigma_{1}$, impulsive noise standard deviation $\sigma_{2}$, impulsive noise occurrence probability $p_{r}$. Note that the parameters of the SGMCC algorithm in Figure 4 are set as α=3, λ=0.25, θ=0.9 and η=0.01.

The comparisons of the theoretical and simulated value on EMSE versus $\sigma_{1}$, $\sigma_{2}$ and $p_{r}$ are shown in Figure 4a–c, respectively. As observed in Figure 4a, the simulated EMSEs match well the theoretical EMSEs computed by Equation (47) and the simulation value grows with the increase of the parameter $\sigma_{1}$. As one can see from Figure 4b,c, the simulated EMSEs also match well the theoretical EMSEs. In Figure 4b, the EMSE values show a very slow downward trend and no significant changes with the increase of the parameter $\sigma_{2}$ from 10 to 60 and from 60 to 100, respectively. In Figure 4c, the EMSE values keep almost the same value with the range of the parameter $p_{r}$ value from 0.02 to 0.2. This implies that the steady-state behavior analysis presented in Section 3.3 is valid and the proposed SGMCC algorithm is robust against impulsive noise.

## 5. Conclusions

In this paper, we propose a smoothed GMCC algorithm called SGMCC to improve the performance of steady-state MSD in the presence of impulsive noise. To avoid the high steady-state MSD caused by the stochastic gradient, instead of taking the exponential weighted average of gradient vector to approximate the expectation of the gradient vector, we take the exponential weighted average of the variable step-size so that the SGMCC algorithm can be viewed as a sign GMCC algorithm with smoothed variable step-size. Convergence performance analyses are derived to demonstrate the robustness of the proposed algorithm and it is verified by the previous simulation part. Simulation results also demonstrate that the proposed SGMCC algorithm is robust against impulsive noise and converges fast with lower computational complexity.

## Figures and Tables

**Table 1:** Comparison of computational complexity.

Algorithm	Computational Complexity
CMCC [4]	6M+10
VSSA [16]	5M+4
GMCC [21]	2M+α+4
SGMCC	2M+α+6

**Table 2:** Parameters setting.

Algorithm	Parameters
CMCC [4]	λ=0.5, $\mu_{a}=4.5$, β=0.8, γ=1.5, σ=2 $\mu_{1}=0.05$, $\mu_{2}=0.01$, $\varepsilon_{1}=\varepsilon_{2}=0.00001$
VSSA [16]	α=0.95, β=0.97, γ=0.0005
GMCC [21]	α=3, λ=0.25, η=0.05
SGMCC	α=3, λ=0.25, η=0.05, θ=0.9
